# Ultra-Processed Food Consumption Associated with Incident Hypertension among Chinese Adults—Results from China Health and Nutrition Survey 1997–2015

**DOI:** 10.3390/nu14224783

**Published:** 2022-11-11

**Authors:** Ming Li, Zumin Shi

**Affiliations:** 1 Centre for Population Health Research, Division of Health Sciences, University of South Australia, Adelaide, SA 5005, Australia; 2 Human Nutrition Department, College of Health Sciences, QU Health, Qatar University, Doha 2713, Qatar

**Keywords:** ultra-processed food, incident hypertension, adults, China

## Abstract

Objective: Ultra-processed food (UPF) has been shown to increase the cardiometabolic health risks. We aimed to determine the association between UPF intake based on the NOVA classification and the risk of hypertension incidence during 1997–2015. Methods: Data from 15,054 adults aged ≥ 20 years (47.4% males) attending the China Nutrition and Health Survey (CNHS) were used. Food intake at each survey was assessed by a 3-day 24 h dietary recall and weighed food record method between 1997–2011. Cox regression was used to assess the association between UPF intake and incident hypertension. Results: During a mean average of 9.5 years (SD 5.5) of follow up, 4329 hypertension incident cases were identified. The incident rates (per 1000) for non-consumers and 1–49, 50–99, and ≥100 g/day of UPF intake were 29.5 and 29.5, 33.4, and 36.3, respectively. Compared with non-consumers, the hazard ratios (95% CI) for UPF intake of 1–49, 50–99, and >100 g/day were 1.00 (0.90–1.12), 1.17 (1.04–1.33), and 1.20 (1.06–1.35), respectively, (*p* = 0.001) after adjusting for potential confounding factors. There was a significant interaction between UPF intake and age with a higher risk in the younger group (<40 years) than in the older one. Conclusion: UPF consumption was dose-responsively associated with increased risk of hypertension among Chinese adults, especially in younger groups.

## 1. Introduction

Hypertension is a serious medical condition that significantly increases the risks of heart, brain, and kidney conditions, as well as other diseases. It is the leading preventable risk factor for cardiovascular disease (CVD) and all-cause mortality worldwide [1]. The global prevalence of hypertension in adults aged 30–79 reached 32% in women and 34% in men in 2019 with an increased trend in most low- and middle-income countries [2], while in China, a review of 15 recent epidemiological studies based on national population surveys from 1997–2017 reported that 18–45% of the Chinese adult population (≥18 years of age) had hypertension, and only a limited portion of 4.2–30.1% had it under control [3].

The sharp increasing trend of hypertension, particularly in younger adults, is in line with the dramatic social–economic development observed in China and multidimensional levels of factors associated with hypertension, including environmental, psychosocial, lifestyle, and behavioral factors [4,5,6,7,8,9].

Among the modifiable dietary factors, certain nutrients, foods, and dietary patterns are associated with high blood pressure/hypertension. For example, high salt consumption has been proven to increase the risk of hypertension substantially in the Chinese population [9]. A meta-analysis of 133 randomized control studies in diverse populations reported that a reduction in sodium decreases systolic blood pressure (SBP) [10]. A recent large 5-year intervention study in Chinese older adults found that using a salt substitute of 70% sodium chloride and 25% potassium chloride decreases SBP and incidence of stroke, CVD, and death, as compared to the use of regular salt [11]. High sodium intake increases blood pressure by increasing water retention and systemic peripheral resistance, altering the endothelial function and the structure and function of large elastic arteries. High intake can modify sympathetic activity, and autonomic neuronal modulation of the cardiovascular system. In addition, excessive dietary sodium induces alterations in the extracellular matrix of the arterial wall, favoring a process of arterial stiffening [12]. World Health Organization recommends limiting sodium intake to approximately 2.0 g per day (equivalent to approximately 5.0 g salt per day) in the general population [13].

Hypertension is inversely associated with intakes of whole grains, fruits, nuts, and dairy, whereas positively with red meat, processed meat, and sugar-sweetened beverages [14] while in the short term, green tea could lower blood pressure [15]. Overall dietary patterns, such as the Dietary Approaches to Stop Hypertension (DASH) study and both the Nordic diet and Mediterranean diet, are associated with blood pressure [16,17]. Studies in the Chinese population have shown that modern dietary pattern with a high consumption of meat and processed foods is associated with increased cardiometabolic risk [18] and DASH diet can reduce the risk of hypertension induced by air pollution [19].

NOVA classifies foods and drinks based on their processing status into four groups, which allows a novel insight into its health impact [20]. Ultra-processed food (UPF) is the 4th group by this classification that includes products of entirely industrial formulations or made from substances extracted from foods, with minimal whole foods [21]. UPF is commonly high in energy density, sugars, salt, and trans-fats, as well as additives with poor nutrition profiles [20], and it contributes more than half of the total daily energy intake in high-income countries, and its consumption is increasing rapidly in middle-income countries [22,23]. The increased consumption was driven by economic development and urbanization, especially in nutrition transition countries, such as China [24,25,26]. In addition, food choice at the individual level based not only on nutrients profile but also on taste, convenience, and cost drives the increased trend [27]. Syntheses of observational studies from countries in Europe and the American continents have shown that UPF intake is associated with certain conditions but the association with hypertension is inconsistent [28,29,30]. For example, the prospective analyses in Mediterranean and Brazilian cohorts demonstrated higher consumption was positively related with the risk of developing hypertension [31,32,33] while results in Canadian and Lebanon adults showed no evidence of a relationship between UPF consumption and SBP and diastolic blood pressure (DBP) [30].

The mean daily UPF consumption in Chinese adults increased four times between 1997–2011, and higher long-term UPF consumption is associated with increased risk of being overweight/obese and diabetes [34,35]. However, the association between UPF consumption and incident hypertension has not been quantified in China and whether the association interplays with being overweight/obese, having diabetes, dietary patterns, or other behavioral factors remains unknown. This study aimed to fill the knowledge gaps.

## 2. Research Design and Methods

### 2.1. Study Design and Sample

This was a prospective follow-up study of UPF intake and incident hypertension between 1997–2015 using data from China Health and Nutrition Survey (CHNS).

**Figure 1:**
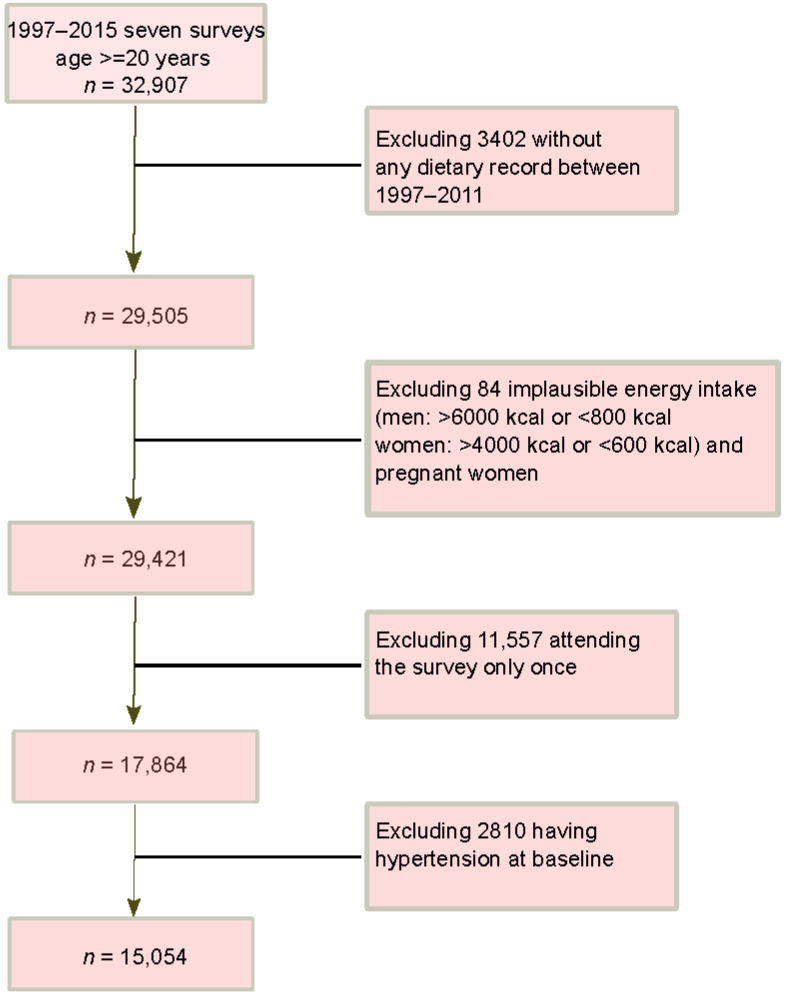
Sample flowchart of participants attending CHNS 1997–2015.

The CHNS study is an ongoing household-based cohort study conducted in nine provinces in China [36]. A multistage random-cluster sampling method was applied to select participants in both urban and rural areas. Ten waves of dietary data collection (1989, 1991, 1993, 1997, 2000, 2004, 2006, 2009, 2011, and 2015) have been completed. The overall response rate was >60% based on the first survey in 1989 and >80% based on the previous survey year [36]. A cohort of 15,054 participants meeting the following inclusion criteria were included (Figure 1): aged ≥ 20 years; having attended at least two nutrition surveys between 1997–2015; having dietary and blood pressure measures; having plausible energy intake (800–6000 kcal/day for men, and 600–4000 kcal/day for women); being free of hypertension at baseline. The survey was approved by the institutional review committees and informed consent was obtained from all participants [36]. The data used in the current study were de-identified and publicly available.

### 2.2. Outcome Variable: Incident Hypertension

During household visit at each survey, blood pressure was measured by mercury sphygmomanometer based on a standard protocol [36]. Hypertension was defined as having SBP ≥ 140 mmHg and/or DBP ≥ 90 mmHg or having known hypertension.

### 2.3. Exposure Variable: UPF Consumption

At each survey, individual food intake data were collected by a trained investigator using a 24 h dietary recall for three consecutive days [36]. Foods and condiments in the home inventory, foods from markets or from gardens, and food waste were weighed and recorded by interviewers at the beginning and end of the three-day survey period. The Chinese food composition tables were used to convert food intake to nutrient intake [37,38]. Around 3000 food items in the food composition tables since 1997 were categorized into four groups based on the NOVA classification [20]. UPF intake for each participant at each survey was categorized into four levels: non-consumers, 1–49 g/day, 50–99 g/day, ≥100 g/day. We choose this cut-off based on the fact that the serving size in the context of Chinese food is *Liang* (50 g).

### 2.4. Covariates

Sociodemographic information was collected at each survey using a structured questionnaire. The following constructed variables were used as indicators of socioeconomic status: education (low: illiterate/primary school; medium: junior middle school; high: high middle school or higher), per capita annual family income (recoded into tertiles as low, medium, and high), urbanization levels (recoded into tertiles as low, medium, and high).

Lifestyle factors from questionnaire included smoking, alcohol drinking, sleep, and physical activity. Smoking status was categorized as non-smokers, ex-smokers, and current smokers. Alcohol consumption was recorded as yes or no. Sleep duration was recorded as ≤6, 7–9, and ≥10 h per day using data collected since 2004. Physical activity level (metabolic equivalent of task MET) was estimated based on self-reported activities (including occupational, domestic, transportation, and leisure time physical activity) and duration using a compendium of physical activities. Tea consumption in each survey wave was categorized into four levels: non-consumers, <2 cups/day, 2–3.9 cups/day, and ≥4 cups/day with one cup being 240 mL.

Height was measured without shoes to the nearest 0.2 cm using a portable stadiometer. Weight was measured without shoes and in light clothing to the nearest 0.1 kg on a calibrated beam scale. Body mass index (BMI) was calculated from weight and height. Overweight/obesity was defined as BMI ≥ 25 kg/m^2^.

### 2.5. Statistical Analysis

Sample characteristics were presented and compared by baseline UPF categories of “None, 1–49, 50–99, ≥100 g/day” using ANOVA for continuous measures or chi-square tests for categorical ones.

The association between UPF intake and incident hypertension was examined using Cox regression with age as the underline time scale [39]. Study entry was the age at baseline. Exit time was the age at incident hypertension or related death or the end of follow-up, whichever occurred first. The proportional hazards assumption was assessed by Schoenfeld residuals. Unadjusted and adjusted hazard ratios (95% CI) were reported from the following models: unadjusted model; adjusted models subsequently adjusted for age, sex, and energy intake; socioeconomic status (income, urbanization, and education), behavioral factors (smoking, alcohol drinking, and physical activity), and BMI; sodium/potassium; intake of fruit and vegetable or green tea; diabetes. All adjusted covariates except sex were treated as time varying measures.

Interaction between UPF intake and other covariates (sociodemographic) on incident hypertension was assessed by introducing a product term in the final regression model (Model 3) and the stratified results were presented. The following sensitivity analysis was conducted: (1) using data from those entering at the first wave (1997) or last wave (2011); (2) data before and after 2004, where UPF increased differently. STATA 17.0 (Stata Corporation, College Station, TX, USA) was used for all the analyses. Statistical significance was considered when *p* < 0.05 (two-sided).

## 3. Results

### 3.1. Population Characteristics and UPF Consumption

Among the 15,054 participants included in this study, 6924 entered in 1997, 2160 in 2000, 1406 in 2004, 774 in 2006, 1320 in 2009, and 2470 in 2011. At baseline, the mean age of this sample was 40.2 years (SD 14.4), 47.4% were males, 40.7% resided in highly urbanized area, 29.7% were smokers, and 8.6% were alcohol drinkers. The prevalence of overweight/obesity was 20.1%. The mean daily energy, fat, protein, and carbohydrate intake were 2184 kcal, 67.8 g, 67.9 g, and 321.9 g, respectively.

At baseline, 11,010 (73%) reported no UPF intake, while 1, 276 (8%) reported daily UPF consumption ≥100 g. Compared with non-consumers, those having ≥100 g/day were significantly more likely to be: older aged; males; having higher education and income; living in highly urbanized area; smoking; drinking; having less tea consumption; sleeping <6 h; having less physical activity; having higher intake of energy, fat, protein, and potassium but lower carbohydrates; having higher fruit intake; entering the survey in a more recent survey; and higher prevalence of overweight/obesity. Baseline prevalence of diabetes were no different by levels of UPF intake (Table 1).

The mean daily UPF consumption in this population increased slowly from 10.5 g in 1997 to 14.9 g in 2004, and sharply increased to reach 47.3 g in 2011 (Supplementary Figure S1).

### 3.2. Incident Hypertension and the Association with UPF Consumption

During a mean average of 9.5 years (median 8.9, SD 5.5) of follow-up (total 142,868 person years), 4329 incident cases were observed. Of them, 689 cases were identified in 2000, 874 in 2004, 575 in 2006, 758 in 2009, 546 in 2011, and 887 in 2015.

The corresponding incident cases for UPF non-consumers, 1–49 g/d, 50–99 g/d, and ≥100 g/d were 3137, 459, 327, and 406, given the unadjusted hazard ratios (HRs) (95% CI) of 1.00, 0.95 (0.86–1.05), 1.08 (0.96–1.21), and 1.12 (1.01–1.25) (*p* for trend = 0.031). After adjusting for age, sex, total energy intake, education, income, urbanization, smoking, alcohol drinking, physical activity, and BMI, the HRs were not substantially changed, being 1.00, 1.00 (0.90–1.12), 1.17 (1.04–1.33), 1.20 (1.06–1.35) (Model 2, Table 2). Further adjusting for sodium/potassium (Model 3), intake of fruit and vegetable/tea (Model 4), or diabetes (Model 5) did not alter the HRs either.

Other factors significantly associated with incident hypertension were age, sex, education, income, urbanization, alcohol drinking, and BMI.

**Figure 2:**
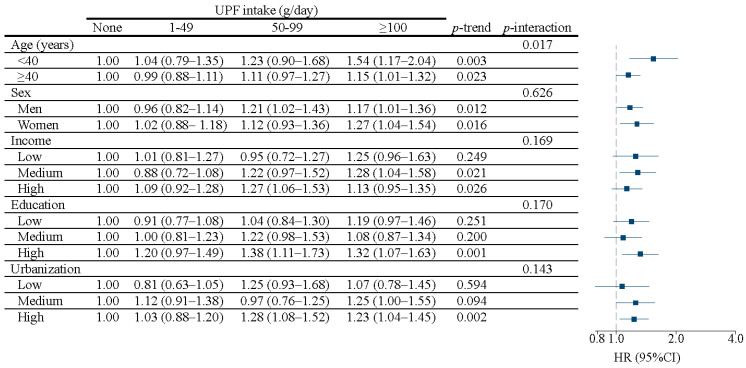
Hazard ratio (95%CI) for hypertension with UPF intake stratified by age, sex, income, education, and urbanization among participants attending China Health and Nutrition Survey (*n* = 15,054). Model adjusted for age, sex and energy intake, income, education, urbanization, smoking, alcohol drinking, physical activity, and BMI. Stratification variables were not adjusted in the corresponding models.

The association between UPF and incident hypertension varied by age. Among the younger participants (<40 years), the adjusted HRs (9% CI) were: 1.04 (0.79–1.35) for 1–49 g/d, 1.23 (0.90–1.68) for 50–99 g/d, and 1.54 (1.17–2.04) for ≥100 g/d, compared to non-consumers, significantly higher than in older participants (≥40 years), with corresponding HRs (95% CI) of 0.99 (0.88–1.11), 1.11 (0.97–1.27), 1.15 (1.01–1.32) (*p* for interaction = 0.017) (Figure 2). There were no significant interactions between UPF and sex, income, education, and urbanization, in relation to the risk of incident hypertension. Sensitivity analysis showed consistent associations (data not shown).

## 4. Discussion

In a 10 year follow-up study of 15,054 adults aged ≥ 20 years, UPF consumption was dose-responsively associated with incident hypertension and those having ≥ 100 g/d had an overall increased risk of 15%. There was a significant interaction between UPF and age. In adults aged under 40 years, high UPF intake (≥100 g/d) increased the risk of hypertension by 54% while there was a 15% increased risk in those aged over 40 years.

Our finding of the positive association between UPF intake and hypertension was consistent with three longitudinal studies: the 9-year follow-up Spanish The Seguimiento Universidad de Navarra Project, project, which reported a 21% higher risk among 14,790 university students [31]; the ELSA-Brazil studies among 8754 adults aged 35–74, which reported 23% greater risk of developing hypertension for higher UPF consumption after adjusting for sociodemographic, lifestyle, BMI, and dietary factors [32]; and the 2-year follow up of 1221 graduates in the Cohort of Universities of Minas Gerais, Brazil (CUME Project) Project that reported an increased risk of 35% [33]. Our study confirmed the results of a meta-analysis of prospective association between certain UPF, such as red meat, processed meat, and sugar-sweetened beverages with hypertension [14].

The positive association between UPFs and hypertension can be explained not only by their poor nutrient profile, including high amount of salt, saturated fats, sugar, and energy, but also a lack of whole foods, such as fruits and vegetables [22,24], which were shown in our adjusted model. Plausible biological pathways may include increased energy intake, changes to the gut microbiota, alterations in the gut–brain satiety signalling, and hormonal effects, which may target sodium/potassium balance, endothelial function, oxidation stress, and inflammation [40]. Despite lacking evidence of the long-term effect of non-nutritional bioactive compounds in UPF on human health and food additives, such as artificial sweetener, emulsifiers, thickening and stabilizing agents, and bisphenols, may play roles through the pathways of insulin response or gut microbiota, and/or adipocyte function [41].

In addition to the poor nutrient profile or quality from UPF that poses a risk to health, such as hypertension, growing concerns have emerged with regard to the impact on the food structure characteristics or food matrix during food processing as UPF products are industrial formulations manufactured from substances extracted from foods or synthesized from other organic sources that mostly contain little or no natural complex food [42,43]. Further research is needed to understand the proportional harm associated with the food physical structure, and other attributes of UPF [44].

The impact of UPF intake ≥100 g/day on the risk of developing hypertension among younger adults is of concern. Based on a previous report using CHNS data, the weekly frequency of eating out doubled to 25% between 2004–2011, remarkably higher in younger adults and males [45]. Eating out increases the consumption of UPF, compared with home-prepared meals [46]. Younger adults are heavily exposed to TV advertisements with more than half on food, snacks, and beverages during the times between 20:00 h to 22:00 h [47]. In addition to these environmental changes, it should be noted that younger adults are under pressure from education, jobs, finance, and family. A recent national survey estimated that 16.6% of Chinese adults had experienced mental illness at some point in their lives with the most common being anxiety disorders and the increased prevalence of depression [48]. Further investigation on the UPF consumption and health transition from childhood and adolescence to adulthood is warranted based on our findings that children and adolescents are more likely to have certain UPF that related to being overweight/obese [49] and to the early onset of hypertension in this study population, in addition to the early onset of some cancers, such as colorectal and breast cancers [50]. It is unknown whether early exposure to UPF or its accumulative effect or both can explain the age difference in association with the early onset of hypertension with UPF.

Our result support the Chinese dietary guidelines published in 2022 in which new recommendations have been supplemented. The new guideline emphasizes the needs to avoid UPF, to acquire knowledge and skills to cook, and to select packaged food by reading food labels, in addition to the food-based recommendations [51].

This is the first association study between UPF consumption using NOVA classification and incident hypertension in a large cohort of the Chinese adult population. The study period lasted for ten years and covered the socioeconomic transition, including dietary pattern change. The energy and food intake from the surveys have been proven to be generally valid based on basal metabolic rate [52]. Missing data were low, and no data imputation was needed. In total, 98.6% of the participants were included in the full multivariable model. Hypertension incident cases were ascertained by established international criteria from data using standardized protocols at each survey. Known confounding factors, including sociodemographic, behavioral, health, and dietary factors were adjusted. The consumption of fat, fruit, and vegetables was used as a proxy for diet quality. The statistical analysis was robust, considering the repeated measures of UPF during the follow-up period and using age as a time scale to reduce potential bias [39].

Limitations should be noted. Firstly, misclassification was possible due to incomplete records on food processing methods in the CHNS survey, which was not specifically matched with NOVA classification, and the use of gram for UPF might not be precise for the diverse UPF items (e.g., soft drinks). Secondly, the ascertainment of food items might not be subtle in reflecting the complexity of food processing and variabilities in additive composition between brands for a similar type of product, and, therefore, some food items could only be roughly grouped and the association could be biased. Thirdly, 24 h sodium excretion, which more accurately measures the dietary sodium and metabolism, was not collected in the CHNS but instead we used dietary sodium in the adjusted analysis. Finally, residual confounding was still possible due to the lack of data on ethnicity, which is closely related to culinary culture in China. In addition, stress level as a strong factor of hypertension was not attainable, although daily sleep and alcohol drinking were included as proxies. Further well-designed studies in other populations and settings are warranted to determine causality and identify potential mechanisms.

To conclude, higher UPF consumption was dose-responsively associated with incident hypertension, especially among younger adults aged < 40 years in a 10-year follow-up of Chinese adults between 1997–2011.

## Figures and Tables

**Table 1:** Baseline sample characteristics by UPF intake (g/day): China Health and Nutrition Survey (*n* = 15,054).

UPF Intake Level	None	1–49	50–99	≥100	*p*-Value
*n*	11,010	1699	1069	1276	
**Survey year**					<0.001
1997	51.8%	41.1%	25.4%	19.4%	
2000	16.1%	11.1%	10.2%	7.0%	
2004	10.3%	8.1%	6.8%	4.5%	
2006	5.0%	5.5%	5.1%	6.0%	
2009	7.7%	9.1%	12.4%	14.5%	
2011	9.0%	25.3%	40.0%	48.7%	
**Age (years), mean (SD)**	39.9 (14.4)	40.8 (14.7)	40.7 (14.4)	41.6 (14.3)	<0.001
**Sex**					<0.001
Men	46.2%	44.2%	49.8%	60.4%	
Women	53.8%	55.8%	50.2%	39.6%	
**Income**					<0.001
Low	31.4%	21.5%	19.4%	17.9%	
Medium	34.5%	32.3%	33.1%	29.4%	
High	34.1%	46.2%	47.5%	52.7%	
**Education**					<0.001
Low	41.1%	27.3%	20.8%	19.8%	
Medium	35.5%	34.2%	29.7%	27.9%	
High	23.4%	38.5%	49.5%	52.2%	
**Urbanization**					<0.001
Low	36.1%	19.3%	15.1%	12.2%	
Medium	30.7%	23.8%	23.2%	21.9%	
High	33.2%	56.9%	61.7%	65.9%	
**Energy intake (kcal/d), mean (SD)**	2206.6 (653.5)	2037.3 (635.2)	2104.3 (670.7)	2260.7 (722.8)	<0.001
**Fat intake (g/d), mean (SD)**	65.4 (35.9)	68.7 (34.0)	75.8 (37.1)	81.3 (38.7)	<0.001
**Protein intake (g/d), mean (SD)**	66.8 (22.7)	67.3 (22.8)	71.2 (23.9)	75.3 (25.6)	<0.001
**Carbohydrate intake (g/d), mean (SD)**	337.2 (125.0)	285.0 (120.8)	275.5 (117.0)	277.7 (107.4)	<0.001
**Sodium intake (mg/d), mean (SD)**	5465.8 (6880.9)	5157.6 (4455.0)	4885.6 (4721.0)	5450.1 (5236.1)	0.014
**Potassium intake (mg/d), mean (SD)**	1611.6 (895.8)	1596.7 (664.2)	1703.7 (757.2)	1872.7 (1174.8)	<0.001
**Vegetable intake (g/day), mean (SD)**	283.7 (173.4)	262.9 (160.2)	253.9 (156.9)	255.3 (158.2)	<0.001
**Fruit intake (g/day), mean (SD)**	20.9 (78.0)	49.7 (103.4)	64.9 (112.8)	89.0 (129.2)	<0.001
**Tea intake (cup/day)**					<0.001
None	64.4%	57.1%	54.4%	51.3%	
<2	12.3%	16.0%	16.0%	17.2%	
2–3.9	12.0%	13.0%	14.6%	12.1%	
4	11.2%	13.8%	15.1%	19.4%	
**Smoking**					<0.001
Non-smoker	69.8%	70.7%	65.0%	60.1%	
Ex-smokers	1.3%	1.4%	3.2%	3.8%	
Current smokers	29.0%	27.8%	31.8%	36.1%	
**Alcohol drinking**	31.7%	35.8%	44.1%	51.5%	<0.001
**Sleep duration (hours/day)**					<0.001
≤6	7.5%	10.3%	9.9%	11.2%	
6–9	80.4%	81.0%	79.6%	80.8%	
>9	12.1%	8.8%	10.5%	8.0%	
**Physical activity (MET hours/week), mean (SD)**	142.3 (115.2)	127.3 (106.2)	127.8 (104.9)	128.4 (99.7)	<0.001
**BMI (kg/m^2^), mean (SD)**	22.3 (3.1)	22.7 (3.2)	22.9 (3.4)	23.2 (3.4)	<0.001
**Diabetes**	5.5%	7.6%	4.7%	8.6%	0.43

*p* from ANOVA for continuous measures or chi-square tests for categorical ones.

**Table 2:** Hazard ratio (95%CI) for hypertension incidence by UPF intake (g/day): China Health and Nutrition Survey (*n* = 15,054).

UPF Intake Level	None	1–49	50–99	≥100	*p* for Trend
Number of incident cases	3137	459	327	406	
Rate (per 1000 person years)	29.5	29.5	33.4	36.3	
Person years	106,364	15,542	9777	11,186	
Unadjusted model	1.00	0.95 (0.86–1.05)	1.08 (0.96–1.21)	1.12 (1.01–1.25)	0.031
Model 1	1.00	1.03 (0.93–1.13)	1.14 (1.02–1.28)	1.25 (1.13–1.39)	0.000
Model 2	1.00	1.00 (0.90–1.12)	1.17 (1.04–1.33)	1.20 (1.06–1.35)	0.001
Model 3	1.00	1.00 (0.90–1.12)	1.17 (1.04–1.33)	1.20 (1.06–1.35)	0.001
Model 4	1.00	1.00 (0.90–1.12)	1.17 (1.03–1.32)	1.19 (1.06–1.34)	0.001
Model 5	1.00	1.00 (0.90–1.12)	1.17 (1.03–1.33)	1.19 (1.06–1.35)	<0.001

Model 1 adjusted for age, sex, and energy intake. Model 2 further adjusted for income, education, urbanization, smoking, alcohol drinking, physical activity, sleep duration, and BMI. Model 3: model 2 further adjusted for sodium/potassium intake. Model 4: model 2 further adjusted for intake of fruit and vegetables/tea; Model 5: model 4 further adjusted for known diabetes.

## Data Availability

The current research uses data from the China Health and Nutrition Survey (CHNS). Data described in the manuscript, code book, and analytic code are made publicly and freely available without restriction at https://www.cpc.unc.edu/projects/china accessed on 15 January 2019.

## Supplementary Materials

The following supporting information can be downloaded at: https://www.mdpi.com/article/10.3390/nu14224783/s1, Figure S1: Age- and sex-adjusted mean intake of UPF in 1997-2011 (*n* = 15,054).
