# Supplementary figures and images for: Ultra-Processed Food Consumption Associated with Incident Hypertension among Chinese Adults—Results from China Health and Nutrition Survey 1997–2015
Source: Nutrients. 2022 Nov 11;14(22):4783. doi: 10.3390/nu14224783 (PMC9692874; doi:10.3390/nu14224783)

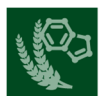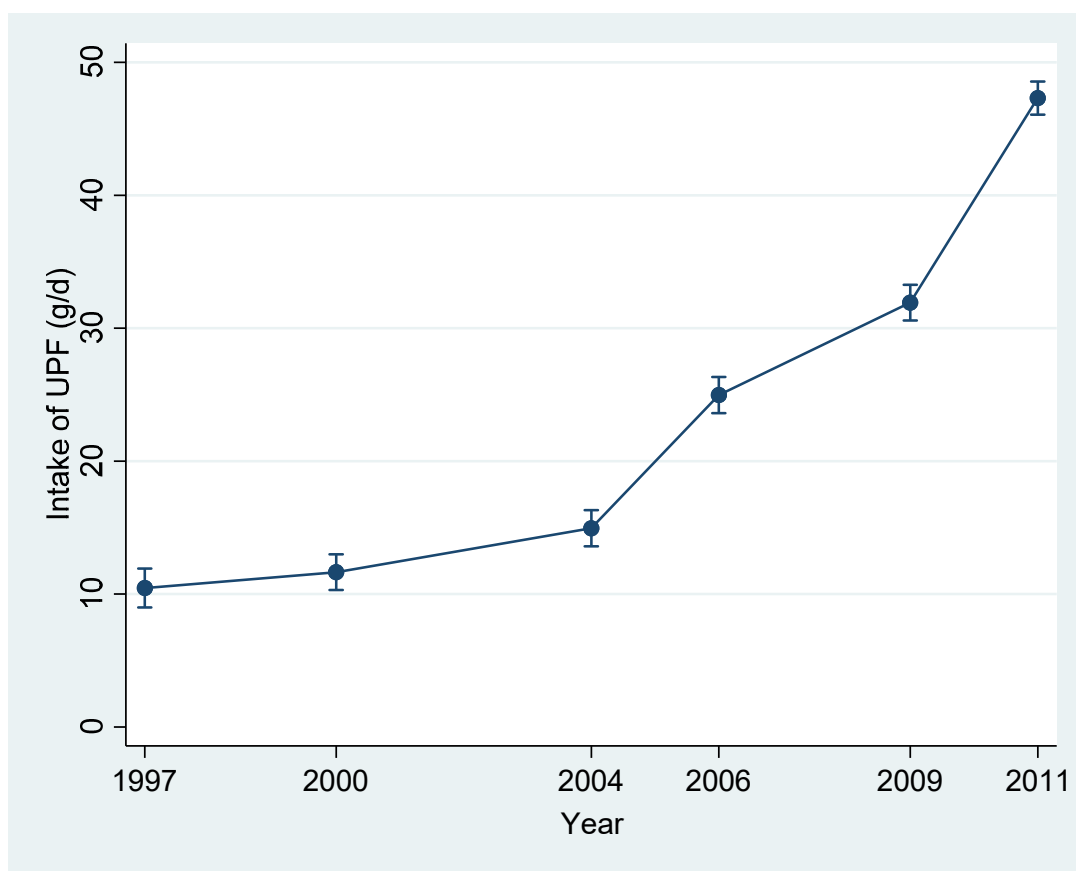

**Supplementary Figure S1.** Age- and sex-adjusted mean intake of UPF in 1997-2011 ( $n = 15,054$ ).

Supplement: Supplementary file 1 [file nutrients-14-04783-s001.zip › nutrients-2025253-supplementary.pdf]
